# Mental health and psychosocial support programmes for displaced populations in low- and middle-income countries (LMICs): A systematic review of process, perspectives and experiences

**DOI:** 10.1017/gmh.2024.56

**Published:** 2024-05-06

**Authors:** Kelly Dickson, Sum Yue (Jessica) Ko, Celine Nguyen, Dayana Minchenko, Mukdarut Bangpan

**Affiliations:** 1 The Evidence for Policy and Practice information and Co-ordinating Centre (EPPI-Centre), Social Research Institute, University College London, London, UK; 2 Southwestern Medical School, University of Texas, Dallas, TX 75390, USA

**Keywords:** mental health, displacement, low income countries, global mental health delivery

## Abstract

Displacement exerts an ongoing negative impact on people’s mental health. The majority of displaced populations are hosted in the global south, yet there is a paucity of evidence synthesis on the implementation of mental health and psychosocial support (MHPSS) programmes in those contexts. We undertook a systematic review of factors influencing the delivery and receipt of MHPSS programmes for displaced populations in low- and middle-income countries to address this gap. A comprehensive search of 12 bibliographic databases, 25 websites and citation checking were undertaken. Studies published in English from 2013 onwards were included if they contained evidence on the perspectives of adults or children who had engaged in, or programmes providers involved in delivering, MHPSS programmes. Fifteen studies were critically appraised and synthesised. Studies considered programme safety as a proxy for acceptability. Other acceptability themes included stigma, culture and gender. Barriers to the accessibility of MHPSS programmes included language, lack of literacy of programme recipients and location of services. To enhance success, future delivery of MHPSS programmes should address gender and cultural norms to limit mental health stigma. Attention should also be given to designing flexible programmes that take into consideration location and language barriers to ensure they maximise accessibility.

## Graphical abstract

**Figure:**
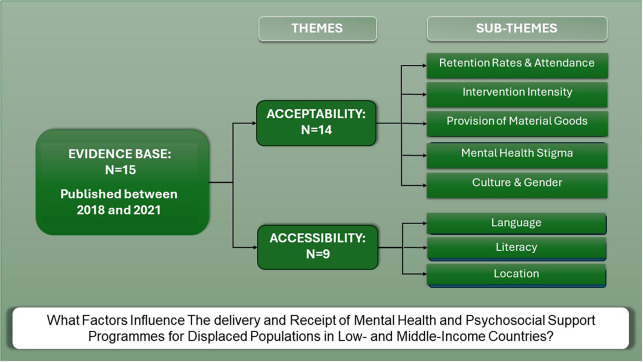

## Impact statement

As the global population continues to experience displacement due to conflict, disasters and other crises, addressing the mental health and psychosocial well-being of displaced communities in low- and middle-income countries (LMICs) remains a critical and ongoing concern. This review has identified 15 high-quality studies on the key factors impacting the acceptability and accessibility of mental health and psychosocial programmes targeting displaced populations in LMICs, such as stigma, gender, language, literacy and the locational reach of services. The stigma surrounding mental health remains pervasive in many societies, impeding help-seeking behaviours and reinforcing a culture of shame around psychological health. Addressing stigma requires psychoeducative approaches that respect cultural beliefs and promote mental health awareness and acceptance. Similarly, acceptability of programme components can differ by gender; thus, an assessment of gender and other sociocultural factors could be assessed during feasibility phases of programme trials to inform whether any adjustments need to be made to ensure greater equity in participation. Consideration of language and literacy barriers is also crucial for ensuring access to all programme components and optimising engagement in MHPSS services. Furthermore, the physical location of the displaced populations can hinder programme accessibility. In remote or conflict-affected regions, access to mental health and psychosocial support services may be limited, and timing and competing demands may necessitate taking a pragmatic approach to programming. Overall, understanding which factors and delivery mechanisms contribute to the successful implementation of MHPSS programmes, prior to scale-up is crucial for ensuring they are inclusive and effective.

## Introduction

The United Nations High Commissioner for Refugees estimated that, by the end of 2022, a total of 108.4 million people were forcibly displaced worldwide. This includes approximately ‘35.3 million refugees, 5.4 million asylum-seekers, 62.5 million internally displaced persons (IDPs) and 5.2 million other people in need of international protection’ (UNHCR, 2023). The majority of individuals and families are hosted in low- and middle-income countries (LMICs), particularly in regions such as Africa, the Middle East and South Asia. The impact of forced displacement on mental health can be significant and long-lasting (Carroll et al., 2023). Displacement can result in a range of psychological distress symptoms, including depression, anxiety, post-traumatic stress disorder and other mental health problems (Patanè et al., 2022). This can be due to a variety of factors, including loss of a person’s home and community, exposure to violence and trauma, uncertainty about the future and limited access to basic needs such as food, shelter and healthcare (Hou et al., 2020). Such factors are often compounded due to the stress that displacement places on an individuals’ social support systems, leading to intensified feelings of isolation, loneliness and psychological distress (Miller and Rasmussen, 2017). Children and adolescents can face distinct challenges, such as prolonged separation from caregivers, risk of exploitation and abuse in unfamiliar and unstable environments and disrupted education. This may lead to psychosocial delays and hinder their long-term development and well-being (Bürgin et al., 2022). The impact of displacement on mental health is often exacerbated by a lack of adequate support, including access to mental health and psychosocial support (MHPSS) services, which can be limited in LMICs. Addressing the mental health needs of displaced populations is therefore crucial to improving their quality of life, well-being and long-term prospects (Sheath et al., 2020).

To shed light on how MHPSS interventions can be improved to better serve displaced populations and their mental health needs, it is paramount that their delivery systems, particularly the ways in which they are implemented, be considered (Nguyen et al., 2023). The successful implementation of MHPSS interventions for populations affected by humanitarian crises is often hindered by a plethora of factors encompassing discrimination, stigma and distrust (Perera et al., 2020; Massazza et al., 2022). More specifically, in the case of displaced populations, challenges can stem from diverse cultural backgrounds and experiences, as well as the adverse experiences they have been exposed to Im et al. (2021).

Primary research and systematic reviews in this area largely assume the form of impact evaluations assessing the effectiveness of MHPSS interventions (Uphoff et al., 2020). While reviews on the delivery and receipt of such interventions remain far fewer, often reflecting the smaller number of primary studies evaluating implementation, particularly in the global south. Given the paucity of evidence synthesis to date, this systematic review aims to fill this gap by answering the following research question ‘*What factors influence the delivery and receipt of MHPSS programmes for displaced populations in LMICs?’* By synthesising data on processes and perspectives, we aim to provide a comprehensive overview of the delivery mechanisms that need to be taken into consideration to ensure successful programme implementation and outcomes.

## Methods

This systematic review was described a priori in a research protocol (Bangpan et al., 2016), and adheres to the Preferred Reporting Items for Systematic Reviews and Meta-Analyses guidance found in Supplementary S1 (Moher et al., 2009).

### Search strategy

We searched 12 bibliographic databases across disciplines and specialist databases: Medline, ERIC, PsycINFO, Econlit, Cochrane Library, IDEAS, IBSS, CINHAL, Scopus, ASSIA, Web of Science and Sociological Abstracts. Both published and unpublished studies were comprehensively searched from the websites of relevant organisations. We searched the citations of included studies and relevant systematic reviews. Search strategies were informed by the scoping exercise (Bangpan et al., 2016) and were developed based on three key concepts (mental health and psychosocial, humanitarian emergencies and study designs). The scoping exercise was instrumental in ensuring we used a comprehensive list of terms for mental health and psychosocial programmes and outcomes that went beyond psychological ‘ill’ health, and terms which could capture implementation data from the perspectives of providers or recipients of MHPSS programmes.

The search was first performed in November 2015 to inform previous reviews on the effectiveness (Bangpan et al., 2019) and barriers and facilitators of delivering MHPSS programmes for people affected by humanitarian crises (Dickson and Bangpan, 2018). This search was updated and finalised in May 2023 to inform this paper (see Supplementary S1 for the example of database search strategies and a list of websites searched).

### Eligibility criteria

To capture evidence that could answer our review question, we included studies published in English from 2013 onwards if they contained qualitative or quantitative data on the delivery and/or receipt of MHPSS programmes for displaced populations affected by humanitarian emergencies in LMICs. To ensure we identified a wide range of interventions, we adhered to the Inter-Agency Standing Committee’s definition of MHPSS and included any programme seeking ‘to protect or promote psychosocial well-being and/or prevent or treat mental disorder’ (Inter-Agency Standing Committee, 2006, p. 11). We defined humanitarian emergencies as natural or man-made emergencies, including both slow-onset and sudden crises, and used the World Bank classification system was used to categorise countries based on their level of economic development (Fantom and Serajuddin, 2016). We took a broad view of displacement to refer to individuals or groups of people who have been forced to leave their homes due to conflict, violence, persecution, natural disasters or other reasons and are unable to return to their communities of origin. This displacement, which often results in different types of settlement status (e.g., status of an individual or group in relation to their residency or citizenship in a particular place or country), could be temporary or permanent. Thus, to guide this review and operationalise displacement, we screened studies using the United Nations Refugee Agency definitions for refugees, asylum seekers and internally displaced populations derived from the 1951 Convention on the Status of Refugees.

Two reviewers (MB and KD) piloted the eligibility criteria. A pilot screening exercise was performed by review team members (MB, KD, CN) before independently screening the studies on titles and abstracts. When there was insufficient information, full reports were obtained to assess the eligibility for inclusion. Double screening was conducted in pairs, on all full texts.

### Data extraction and quality appraisal

A fit-for-purpose data extraction tool was used to capture key dimensions to answer the review question and provide study context details. Key information included the following: bibliographic details, participant and intervention characteristics, study methods and findings (see Table 1). Piloting and refinement of the tools took place before the commencement of full coding. We used the EPPI-Centre quality appraisal tool, appropriate for qualitative and mixed methods studies of process data (Dickson et al., 2018; Dickson and Bangpan, 2018), to determine the trustworthiness of the evidence based according to two key dimensions: reliability and usefulness. Criteria to judge reliability are achieved by evaluating efforts to minimise bias and/or increase rigour in the sampling, data collection, and data analysis processes; and takes into account the studies’ ability to demonstrate how findings and conclusions were derived from the collected data, as well as whether the studies’ findings had achieved breadth (i.e., considered the perspectives of multiple participants) or depth (i.e., if the study made insightful and meaningful contributions to existing literature, concepts or theories). Criteria to judge usefulness in answering the review question take into consideration the reflexivity of the primary study authors, namely, through whether they had considered the power relations between themselves and the participants, and if steps were taken to assure participants of their rights and confidentiality. The approach to determining the overall quality of each study according to each dimension is provided in Supplementary S1. To support quality assurance processes, data extraction and quality appraisal were performed by at least two members of the research team, and any discrepancies were discussed and reconciled with a third member of the team.

**Table 1.** Key characteristics of the intervention and context of delivery

Authors	Displacement context		Intervention details					
	Civil war in	Setting	Programme	Target age	Format	Frequency and intensity	Delivery site	Key components
Akhtar et al. (2021)	Syria	Jordan	Early Adolescent Skills for Emotions (EASE)	Children and adults	Group	Children: Seven 90–min sessions Caregivers: Three 120–min sessions	Unspecified	Child sessions: psychoeducation, problem solving, stress management (diaphragmatic breathing), behavioural activation and relapse prevention. Caregiver sessions: psychoeducation, active listening, quality time, praise, self–care and relapse prevention. EASE was initially adapted for Syrians residing in Lebanon and further adapted for use in Jordan.
Bawadi et al. (2022)	Syria	Jordan	Mental health services in Jordan	All	Individual	Unspecified	Clinic	Physicians are allowed to prescribe psychotropic medications. Public sector primary healthcare physicians are allowed to prescribe psychotropic medications but under certain conditions; they may prescribe follow–up treatment, for example, but cannot initiate treatment. The number of psychiatrists in Jordan does not exceed 2 per 100,000 residents and the number of nurses is 0.04 per 100,000.
Doğan et al. (2019)	Syria	Turkey	Mental health services in Turkey	All	Individual	Unspecified	Hospital	General psychiatric services with the aim of protecting, treating and rehabilitating mental health. Syrian doctors and nurses were granted the “Vocational Certificate of Authority” in November 2016, which enabled these staff members to provide health services to Syrian patients in Turkey.
Doumit et al., 2020	Syria	Lebanon	Creating opportunities for patient empowerment (COPE)	Children	Group	One weekly 60–min session for 7 weeks	Community Centre and text messages	Discussion of thinking–feeling–behaving triangle, including how to: engage in positive self–talk, “stay in the moment”; healthy vs. unhealthy self–esteem, work on enhancing self–esteem; deal with emotions in healthy ways, and communicate effectively. Skill building on how to reduce stress and engage in healthy coping, including how stress affects people and positive ways to deal with stress, overview of signs and symptoms of anxiety and depression. Goal setting, the four steps used in problem solving, and how to overcome barriers in achieving one’s goals. information on mental and guided imagery, and how they could practice it
El–Khani et al. (2021)	Afghanistan	Serbia	Strong Families Programme	Children and Adults	Group	One weekly session for 3 weeks, spanning a total of 5 h per family.	Reception centre	Children sessions: activities taught children how to deal with stress and to observe rules and responsibilities Caregiver sessions: supported caregivers in developing ways to deal with stress and show children care while enforcing appropriate limits. Family sessions allowed caregivers and children to come together to learn to communicate, practise stress relief techniques together and show appreciation for one another. This version of Strong Families was previously culturally adapted, translated and reviewed in Afghanistan, and then translated to Serbian for the purpose of training and implementation in Serbia.
Fine et al. (2021)	Burundi	Tanzania	Early Adolescent Skills for Emotions (EASE)	Children and Adults	Group	Children: A 90–min weekly session for 7 weeks Caregivers: Three 2–h sessions	Unspecified	Child sessions: cognitive behavioural strategies including psychoeducation, stress management, behavioural activation, problem solving and relapse prevention. Caregiver sessions: psychoeducation, active listening, slow breathing, positive parenting strategies, caregiver self–care and relapse prevention. EASE was adapted for Burundian refugee young adolescents and their caregivers, with changes made to ensure that the intervention would be culturally and contextually appropriate.
Greene et al. (2022)	Democratic Republic of the Congo	Tanzania	Nguvu intervention	Adults	Individual and Group	Eight sessions	Refugee camp	An integration of cognitive processing therapy and advocacy counselling. Cognitive processing therapy is a manualised, evidence–based psychotherapeutic intervention developed for survivors of assault that focuses on developing skills to manage distressing thoughts that lead to emotional problems. Advocacy counselling focuses on increasing autonomy, empowerment and strengthening linkages to community services by supporting survivors in exploring potential strategies that are supported by the facilitator through safety planning and goal setting.
Hamid et al. (2020)	Syria	Turkey	Mental health services in Turkey	All	Individual	Unspecified	Skype, WhatsApp, clients’ homes etc.	Provision of therapy by Syrian mental health professionals
Kerbage et al. (2020)	Syria	Lebanon	Mental health services in Lebanon	All	Individual	Unspecified	Clinics	Non–specialised psychosocial support by social workers, as well as specialised services provided by psychotherapists and psychologists.
Miller et al. (2020)	Syria	Gaza, Lebanon	Caregiver Support Intervention (CSI)	Adults	Group	One 2–h weekly session for 9 weeks	Unspecified	A combination of interactive and didactic activities that aim to lower stress and improve psychosocial well–being among parents, as well as improve skills related to positive parenting. In selecting content for the CSI, the authors adopted a culturally integrative approach. For example, they addressed anger and frustration using the Arabic and Turkish concept of Asabi, a salient cultural idiom often used among members of the targeted population. To avoid religious connotations that might cause discomfort in the traditional Muslim communities, they also avoided the term ‘mindfulness’ due to its Buddhist roots, instead referring to all of the stress management activities simply as ‘relaxation exercises’.
Mitchell–Gillespie et al. (2020)	Multiple (i.e., Palestine, Syria, Iraq)	Jordan	Community–based rehabilitation (CBR)	Adults	Individual	Unspecified	CBR Centre	Dissemination of rehabilitation services to individuals with physical and mental disabilities.
Murray et al. (2018)	Somalia	Ethiopia	Common elements treatment approach for youth (CETA–youth)	Children and Adults	Individual child and caregiver pairs	Six to twelve 60– to 90–min weekly sessions, depending on need	Refugee camp	Comprises elements of engagement, psychoeducational, parenting skills (for the caregiver), anxiety management strategies, behavioural activation, cognitive and coping/restructuring, imaginal gradual exposure, in vivo exposure, problem solving, suicide/homicide/danger assessment and planning. All the chosen local counsellors were fluent in Somali.
Powell and Qushua, 2023	Syria	Jordan	The Healthy Community Clinic	Adults	Group	Four sessions	Ministry of Health Clinic	The intervention used a strengths–based approach employing psychoeducation, solution focussed techniques and mindfulness exercises to increase awareness of mental health challenges, and to amplify participants healthy coping strategies. The psychoeducational material provided participants with information on the impact of chronic stress on physical and mental health; how stress manifests in the mind and body; traumatic stress symptoms; and strategies to mitigate or alleviate the impact of chronic or traumatic stress such as social and community support, healthy coping strategies. Solution–focussed activities included small group discussions where participants identified strategies to reduce the impact of stressors such as taking a walk or reaching out to a trusted individual.
Tol et al. (2018)	South Sudan	Uganda	Self–Help Plus (SH+)	Adults	Individual and group	Five 2–h weekly sessions	Refugee camp	A pre–recorded audio course and an illustrated self–help manual, which aim to decrease psychological distress. Facilitators were selected based on their proficiency in Juba Arabic and English, among other criteria.
Yassin et al. (2018)	Palestine	Lebanon	Mental Health Programme	Children and adults	Individual and group	Unspecified	Mental health centre	The programme provided free access to mental health care services, specifically psychiatric/mental health assessment, onsite therapy and prescription of medications.

### Data synthesis

Using an EPPI reviewer, at least two authors extracted key ideas and concepts of each included study pertaining to factors that influenced the delivery of MHPSS interventions. The data analysis was carried out using thematic synthesis (Thomas and Harden, 2008) and drawing on elements of Noblit and Hare meta-ethnographical approach to synthesis (Noblit and Hare, 1988). We chose a hybrid approach to support the integration of both qualitative and quantitative data and ensure we were sensitive to negative case examples and contradictory findings within any given theme. This approach was supported by identifying ‘reciprocal translations’, where concepts extracted across studies were similar and could be incorporated into one another to create higher level themes, as well as ‘refutational translations’, where concepts are similar enough to group together but provide ‘negative case examples’ or contradictory findings (Uny et al., 2017). For the former, common themes emerged as a product of the synthesis, whereas in the case of the latter, we explored the reasons for the contradictions as part of the synthesis. We integrated the identified ‘reciprocal translations’ and ‘refutational translations’ using a ‘line of argument’ synthesis, creating an overall narrative of the delivery and receipt of MHPSS interventions for displaced populations (Brookfield et al., 2019). This narrative informed our recommendations for the future design of such interventions.

## Results

### Search results

**Figure 1.**
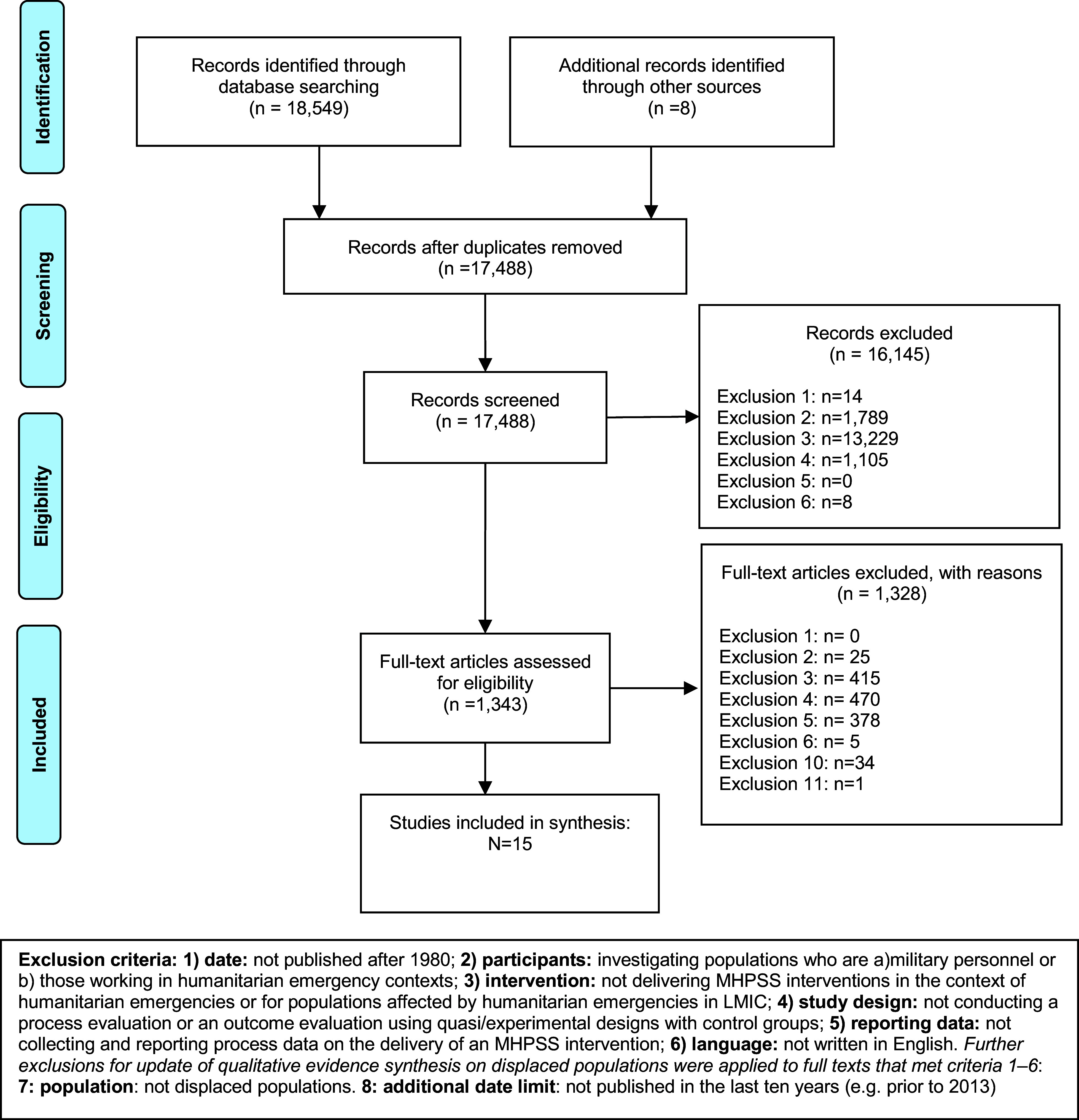
Preferred Reporting Items for Systematic Reviews and Meta-Analyses flowchart.

We identified 18,557 references, in which 17,488 references were screened on the basis of title and abstract, and 1,343 references were rescreened on the basis of the full-text reports. A total of 15 studies were included in the review (see Figure 1 Preferred Reporting Items for Systematic Reviews and Meta-Analyses flowchart).

### Characteristics of studies

An overview of the included studies is provided in table 1 and table 2. Studies were published between 2018 and 2021, with sample sizes ranging between 8 and 85 (see Table 2). The aims of the studies were relatively varied, with a sizeable number examining feasibility, acceptability and accessibility. Study designs utilised across the studies were either of a qualitative (e.g., conducting interviews and focus groups to understand the perceptions and experiences of displaced populations and programme providers) or mixed-methods nature (e.g., also conducted statistical analysis, such as measures of attendance).

**Table 2.** Aims, methods and overall quality of studies

Authors	Study aims	Study design and sample size			Overall quality	
		Study sample	Data collection	Data analysis	Reliability	Usefulness
Akhtar et al. (2021)	To test the safety, feasibility and trial procedures of the EASE intervention among Syrian refugee youth in preparation for a randomised controlled trial.	Recipients: N = 9 Caregivers: N = 9 Facilitators: N = 16	Surveys Focus groups Interviews	Statistical analysis Grounded theory	High	High
Bawadi et al. (2022)	To explore the perspectives of Syrian refugees and their host communities and community leaders in Jordan on barriers and facilitators to the use of mental health services by Syrian refugees.	Recipients: N = 16 Professionals: N = 8	Interviews	Thematic analysis	High	High
Doğan et al. (2019)	To investigate Syrian refugee adults’ experiences with mental health services.	Recipients: N = 24	Focus groups	Phenomenological analysis	High	High
Doumit et al. (2020)	To assess the feasibility, acceptability and preliminary effects of a cognitive–behavioural intervention	Recipients: N = 31	Survey	Descriptive analysis	High	Medium
El–Khani et al. (2021)	To evaluate the feasibility of delivery and any potential impact of Strong Families with refugees.	Caregivers: N = 8 Facilitators: N = 6	Questionnaires Interviews	Statistical analysis Thematic analysis	Medium	Medium
Fine et al. (2021)	To evaluate the feasibility, acceptability, relevance and safety of the Early Adolescent Skills for Emotions (EASE) intervention among Burundian refugee adolescents and their caregivers in Tanzania	Recipients: N = 10 Caregivers: N = 11 Facilitators: N = 15	Interviews	Thematic analysis	High	Medium
Greene et al. (2022)	To examine the relevance, acceptability, feasibility and impact of implementing the Nguvu intervention in a refugee setting from the perspective of intervention stakeholders.	Recipients: N = 10 Facilitators: N = 10 Stakeholders: N = 9	Interviews	Thematic analysis	High	Medium
Hamid et al. (2020)	To explore the impact of the provision of care that forcibly displaced Syrian mental health professionals (MHPs) give to Syrian clients in the community.	Professionals: N = 16	Interviews	Thematic analysis	High	High
Kerbage et al. (2020)	To explore the perceptions and experiences of practitioners, policymakers and Syrian participants involved in mental health services for refugees in Lebanon.	Recipients: N = 25 Facilitators and policymakers: N = 60	Interviews	Thematic analysis	High	High
Miller et al. (2020)	To describe the iterative process by which the authors developed the Caregiver Support Intervention (CSI).	Recipients: N = 48	Focus groups	Thematic analysis	High	High
Mitchell–Gillespie et al. (2020)	To explore the feasibility and acceptability of implementing a telehealth system to support community–based rehabilitation workers in a refugee camp CBR centre in Jordan.	Facilitators: N = 8	Surveys Focus groups	Thematic analysis	Medium	High
Murray et al. (2018)	To evaluate the delivery and implementation of a common elements treatment approach for children in three Somali refugee camps on the Ethiopian/Somali border.	Recipients and Caregivers: N = 35 Facilitators: N = 16	Interviews Pre–post scores	Thematic analysis Statistical analysis	High	Medium
Powell and Qushua (2023)	To examine Jordanian and displaced Syrian participants’ experience attending the Healthy Community Clinic, an integrated mental health awareness intervention delivered in a primary care clinic in Irbid, Jordan.	Recipients: N = 21	Interviews Focus groups	Thematic analysis	High	High
Tol et al. (2018)	To describe the adaptation and piloting of a guided, multimedia, self–help intervention, Self–Help Plus (SH+), for South Sudanese refugees in Uganda.	Recipients: N = 65	Interviews	Statistical analysis Thematic analysis	Medium	Medium
Yassin et al. (2018)	To evaluate the impact of a Mental Health Programme for Palestinian Refugees in Lebanon.	Recipients: N = 28 Facilitators: N = 11 Stakeholders: N = 10	Focus groups	Thematic analysis	High	High

The studies were centred on displaced populations related to the civil war in a range of countries in South Asia (n = 1), Africa (n = 3) and the Middle East (n = 8), with roughly half the studies pertaining to the civil war in Syria (n = 6) (see Table 1). Barring one exception (El-Khani et al., 2021), the respective MHPSS implementation sites of each study was situated in neighbouring countries within the same continent. Among the studies, two categories of MHPSS were recurrent (see Table 1). The first was interventions that sought to support and/or educate displaced caregivers and parents (n = 5), such as Early Adolescent Skills for Emotions (EASE), the Strong Families Programme, Caregiver Support Intervention (CSI) and the common elements treatment approach for youth (CETA-youth). These studies investigated caregiver-child dyads. The second was the provision of general mental health services (n = 4). Cultural adaptation of programmes varied across studies, with some drawing on stakeholders prior to implementation to ensure they were sensitive to local contexts, while other studies considered cultural suitability and the need for adaption as part of the programme evaluation (further details are provided in Supplementary S1).

#### Quality of studies

The findings of this review are grounded in a strong evidence base. Overall, study quality was judged to be of high or medium reliability and usefulness, with none of the studies judged to be of low quality on either dimension (see Table 2). More than half of the studies (N = 9) were judged to have met all of the criteria established by the tool (e.g., they took steps to minimise bias and increase rigour in sampling, data collection and analysis, ensured their findings were grounded in the data, achieved breadth and/or depth in their findings and privileged the views of participants). Of the remaining six studies, four scored at least ‘high’ or ‘medium in at least one of the two dimensions and two studies were judged as medium on both dimensions (see Supplementary S1 for a full breakdown of study quality)

#### Synthesis

An overview of the themes are provided in Table 3.

**Table 3.** Overview of themes and subthemes

Theme	Subthemes	Number of studies
Safety	N/A	N = 4 (Murray et al., 2018; Miller et al., 2020; Akhtar et al., 2021; Greene et al., 2022)
Acceptability	Retention/attendance	N = 6 (Doumit et al., 2020, Miller et al., 2020, Akhtar et al., 2021, Greene et al., 2022), Murray et al., 2018, Tol et al., 2018)
	Intervention Intensity	N = 4 (El–Khani et al., 2021; Fine et al., 2021; Greene et al., 2022; Powell and Qushua, 2023)
	Provision of material goods	N = 3 (Tol et al., 2018; Kerbage et al., 2020; Greene et al., 2022)
	Stigma	N = 6 (Bawadi et al., 2022, Hamid et al., 2020, Kerbage et al., 2020; Mitchell–Gillespie et al., 2020; Powell and Qushua, 2023; Yassin et al., 2018)
	Culture	N = 4 (Murray et al., 2018, Hamid et al., 2020, Kerbage et al., 2020, El–Khani et al., 2021)
	Gender	N = 3 (Miller et al., 2020; Greene et al., 2022; Powell and Qushua, 2023)
Accessibility	Language	N = 4 (Tol et al., 2018, Doğan et al., 2019, Hamid et al., 2020, Mitchell–Gillespie et al., 2020)
	Literacy	N = 3 (Tol et al., 2018, Kerbage et al., 2020, Fine et al., 2021)
	Location/reach	N = 7 (Yassin et al., 2018, Doğan et al., 2019, Mitchell–Gillespie et al., 2020, Akhtar et al., 2021, El–Khani et al., 2021, Fine et al., 2021, Greene et al., 2022)

#### Theme 1: Safety

Programme safety was investigated in three studies (Akhtar et al., 2021; Fine et al., 2021; Greene et al., 2022). Two studies evaluated the feasibility of the EASE intervention; one delivered to Syrian refugees and their caregivers in Jordan (Akhtar et al., 2021) and the other with Burundian refugee adolescents and their caregivers in Tanzania (Fine et al., 2021). The remaining study programme focussed on MHPSS approaches to reduce psychological distress in Congolese refugee women who had experienced intimate partner violence (Greene et al., 2022). All three programmes put safety protocols in place to ensure participants were protected from harm. Greene et al. (2022) took steps to retain the privacy of involvement in the intervention ‘given concerns that this may be unacceptable to their partners and may put women at increased risk of violence’ (pp. 2874–2875). This was achieved by presenting the programme to be about women’s overall health and well-being and selecting community members as facilitators to increase trust. Despite this, ‘some women nevertheless struggled with getting permission from their partners to attend sessions’ (p. 2875). However, overall, no adverse events were made known during the study, and confidentiality was reported as high among both the participants and facilitators. Similarly, no adverse events were reported ‘for children or caregivers’ participating in the Jordan-based EASE programme, ‘including during the screening and assessment phases’. Overall, the trial protocols indicated that ‘appropriate safeguarding of children and identification of those most-at-risk’ (p. 10) were in place. The authors suggested that, this would not have been the case if ‘during the screening, two children were found to be at-risk of suicide’ were not ‘immediately referred to child protection services for appropriate support (Akhtar et al., 2021, p. 10). The safety procedures in the Tanzania-based study by Fine et al. (2021) also ensured that any participants experiencing significant distress and/or violence during the intervention would receive referrals to specialised services. Of the 24 adverse events reported to the Data Safety Monitoring Board, only one was found to be related to the intervention. This was due to a non-participant who was ‘harassing adolescent participants due to tensions over not receiving the EASE sessions’ (p. 8) rather than participating in the intervention itself. To address this, the authors noted a greater need for privacy by ensuring ‘a discreet yet accessible location’ was chosen for programme delivery, particularly for participants who live ‘in close-residing communities such as a refugee camp’ as well as ‘greater geographical distance between the intervention and control conditions, and the implementation of non-therapeutic structured activities’ for participants who had been screened out (Fine et al. p. 9).

#### Theme 2: Acceptability

The acceptability of MHPSS programmes was a key theme in 14 of the included studies (Murray et al., 2018; Tol et al., 2018; Yassin et al., 2018; Doumit et al., 2020; Hamid et al., 2020; Kerbage et al., 2020; Miller et al., 2020; Mitchell-Gillespie et al., 2020; Akhtar et al., 2021; El-Khani et al., 2021; Fine et al., 2021; Bawadi et al., 2022; Greene et al., 2022; Powell and Qushua, 2023). The six sub-themes included: i) retention rates and attendance, ii) intervention intensity, iii) provision of material goods, iv) the stigma associated with mental health, v) culture and vi) gender.

### Retention/attendance

Although many factors can influence programme attendance, such as accessibility and scheduling constraints, attendance rates were mostly used as a proxy for programme acceptability. Five studies collected attendance data to assess programme feasibility and support future implementation strategies (Murray et al., 2018; Tol et al., 2018; Doumit et al., 2020; Miller et al., 2020; Akhtar et al., 2021). For example, the three-phase evaluation by Miller et al. (2020) reports high engagement with the CSI, with no dropouts, and a total of 85% of adult Syrian refugees ‘completing seven or all eight of the sessions’ (p. 5). In phase 2, the rates remained high but fell slightly, with 75% of women and 73% of men attending at least seven of the nine sessions. Furthermore, ‘11 of the 38 men in Lebanon dropped out of the intervention, as did nine of the 36 women’ (p. 6) compared to the Gaza site where there were no dropouts. The reasons for this attrition were explored via interviews and rectified to ensure higher attendance rates in phase 3. Addressing programme content and delivery mechanisms proved to increase the attendance rates to ‘95% of women and 86% of men completing at least seven of the nine sessions’ (pp. 7–8). High attendance rates were also reported by Akhtar et al. (2021), with 78.78% of participants attending at least five out of seven EASE sessions’ (p. 7). When evaluating the CETA for children in three Somali refugee camps on the Ethiopian/Somali border, Murray et al. (2018) found that caregivers were engaged and attended an ‘average of 9.42 sessions out of 13′ (p. 8).

Although favourable satisfaction rates (e.g., a mean rating of 8.54/10.00, SD = 0.13) were reported in the study by Doumit et al. (2020), for ‘unknown reasons’, six Syrian refugees participating in the Lebanon CBT-based programme for ‘were lost to attrition by the second session’ (p. 230). However, an overall retention rate of 77.5% was achieved by the end of the study (N = 31 participants).

In Tol et al.’s (2018) pilot study of Self-Help Plus (SH+), for South Sudanese refugees in Uganda, they found variation in attendance rates among male and female participants. For example, the attendance in the female group was high, with ‘76% of women (n = 25) missing none or one (out of five) sessions, and a further 25% (n = 8) missed two or three sessions’ (p. 6). The attendance ‘in the male group was mixed with 38% of men (n = 12) missing none or one (out of five) sessions, 31% (n = 10) missed two or three sessions, but 31% (n = 10) missing four sessions’ (p. 6); suggesting there were some gender differences related to acceptability (see section 2.6).

### Intervention intensity

Four studies made observations about increasing the intensity and duration of MHPSS interventions. Greene et al. (2022) noted that ‘participants, facilitators, research staff and members of the community advisory board described the desire for continued delivery of Nguvu sessions’ (p. 2873). Resettled Syrians also expressed a strong desire for continued involvement in an integrated mental health awareness intervention delivered in primary care clinics in Jordan (Powell and Qushua, 2023), including the need for more information ‘about mental health, addictions and everything’ (p. 168). Caregivers participating in the Strong Families Programme also ‘expressed an interest in continuing to learn family skills and more involvement’ with several suggesting they did not want the programme to end (El-Khani et al., 2021, p. 14). In the Tanzanian-based EASE study, both adolescents and their caregivers ‘gave positive feedback on the length and frequency of EASE sessions’ but many also found that they ‘would have benefited from more and/or longer sessions (p. 6).

### Provision of material goods

There was a misconception among participants in the three studies that they would receive material and financial aid alongside mental health support. Tol et al. (2018) report that ‘the fact that no basic goods or services were provided as part of SH+ delivery was one of the primary challenges… mentioned by all participants’ (p. 9). This was despite repeated attempts to communicate that mental health was the primary aim of the intervention. ‘Similarly, there were expectations across stakeholder groups that they would be provided with material or financial support related to their different roles and participation’ in the study by Greene et al. (2022). In many cases, participants requested ‘small items (e.g., soap, food) or financial support during Nguvu sessions similar to the compensation they received when completing research interviews’ (p. 2874). Providers of mental health services in the study by Kerbage et al. (2020) commented that participants ‘want a job, material aids, but we tell them we cannot help them materially but psychologically’ (p. 6). The study authors also noted that providers often ‘complained that Syrians repeatedly asked about material aid in therapeutic settings’ making it difficult for them to communicate the value and need to attend to their mental health (p. 7).

### Stigma

Stigma surrounding the need for and use of mental health services was a recurring theme in six studies (Yassin et al., 2018; Hamid et al., 2020; Kerbage et al., 2020; Mitchell-Gillespie et al., 2020; Bawadi et al., 2022; Powell and Qushua, 2023). The studies largely concluded that mental health treatment was associated with negative labels such as the term ‘crazy’ within the investigated contexts making engagement with mental health programmes less acceptable amidst displaced populations (Bawadi et al., 2022, p. 199; Hamid et al., 2020, p. 8; Kerbage et al., 2020, p. 4; Yassin et al., 2018, p. 394). The studies also suggested that MHPSS users would bring shame to, or face rejection from, loved ones and the community due to social stigma. For example, in Yassin et al. (2018), ‘a few patients reported that family members still did not accept their illness, sometimes criticising them’ (p. 394) and women in the study by Bawadi et al. (2022) were less likely to seek mental health support in case it causes ramifications in their relationships. For example, one participant shared that: ‘If my husband knows I have been visiting the mental health clinic he will divorce me’ (female 22 years).

Mitchell-Gillespie et al. (2020) reported the perceived likelihood by community-based rehabilitation (CBR) workers that parents of children undergoing CBR would be reluctant to have CBR sessions filmed for fear that they ‘will be published’ or ‘distributed’ (p. 10). While the participants in Kerbage et al. (2020) ‘did not feel ashamed’ for using mental health services, they had mentally segregated themselves from sufferers of mental illness, regarding their symptoms as ‘a normal and collective reaction to their situation’ (p. 8). Therefore, what appears to be a discrepancy reported by Kerbage et al. (2020) does not contradict the notion that mental illness was associated with shame and rejection. Given the stigma associated with MHPSS and the possible repercussions of using such services, intervention recipients were generally fearful that they would be found out. In Yassin et al. (2018), a participant expressed that they did not ‘want anyone to know’ (p. 394) about them receiving treatment, while in Hamid et al. (2020), it was acknowledged that seeking treatment was ‘often not spoken about’ as people generally ‘do not tell each other that we need help’ (p. 5).

### Culture

Culture was another key factor that influenced the acceptability of mental health interventions among displaced populations (Murray et al., 2018; Hamid et al., 2020; Kerbage et al., 2020; El-Khani et al., 2021). Overall, studies showed that recipients engaged better when MHPSS interventions took into account of the cultural context. For example, in El-Khani et al. (2021), ‘caregivers and facilitators’ deemed the Strong Families intervention to be ‘culturally appropriate’. As one participant reflected “What we learnt (in the intervention) is all in our religion; to respect each other, our families as well and respect the elderly” (p. 13). This led the authors to perceive this as one of the reasons for participants’ ‘engagement and satisfaction’ (p. 13). A similar observation is also noted in Murray et al. (2018), which discusses how ‘clients’ religious and cultural views’ influenced their ‘level of engagement’ (p. 10).

Three studies explicated how and/or why recipients’ religious beliefs impacted their acceptance of MHPSS programmes. In El-Khani et al. (2021), one participant indicated that the Strong Families intervention aligned well with their Islamic beliefs, which place emphasis on ‘family’ and ‘respect’ for the ‘elderly’ (p. 13). Likewise, in Murray et al. (2018), participants were sceptical and considered ‘counselling a somewhat foreign form of treatment’ due to their belief that ‘mental health issues came from a higher religious power’ (p. 10). As such, the most common solution was ‘to have the afflicted parties read religious scripture’ (p. 10). Finally, in Hamid et al. (2020), mental health professionals pinpointed that their understanding of clients’ ‘religious norms and practices’ (p. 4), such as their nuanced comprehension of ‘the male–female relationship in relation to disclosing emotional experiences’ (p. 4), helped them to make appropriate and acceptable decisions for their clients, such as referring male professionals to male clients and vice versa.

Correspondingly, tensions arose when mental health professionals were unable to understand the cultural norms of their clients, which adversely affected professionals’ acceptability of their clients. As illustrated in Kerbage et al. (2020), Lebanese mental health practitioners equated ‘Syrian culture with behavioural ineptitude’, which prevented them from ‘identifying with the displacement experience’ of their clients (p. 11). Further, these practitioners determined Syrian culture to be an ‘obstacle’ (p. 11) to the effective delivery of mental health services given that Syrian refugees do not have the ‘culture for mental health’ and ‘do not see the need for’ or ‘understand why they should come’ (p. 6) to such services.

### Gender

Different responses to programme content and structure along gender lines were highlighted in three studies (Miller et al., 2020; Greene et al., 2022; Powell and Qushua, 2023). Miller et al., 2020 noted that there was a ‘reluctance among men to try the stress management exercises’ without understanding ‘the science behind them’ (p. 7) compared to women’s enthusiasm. This contrasted with male participants’ willingness to engage in frustration and anger management techniques that they ‘viewed quite positively’ (p. 7). Content on early childhood development was also perceived as ‘irrelevant’ by men, who believed that they ‘had essentially no role to play in the raising of children below the age of 5’, with one man suggesting that, ‘from zero to four there is nothing to talk about.’ (p. 7). This differed from the female participants who ‘requested more ‘quality time’ activities they could engage in with very young children, and to have these available in recorded form like the relaxation exercises’ in addition to ‘a written manual with all of the parenting methods and activities learned in the sessions’ (p. 7). In the strengths-based and solution-focussed MHPSS programme evaluated by Powell and Qushua (2023), there were suggestions that gender-separated groups would be preferable. As ‘sometimes there are questions males want to ask but they feel shy and the same with women they want to ask question and they feel shy to ask, so later we ask the questions individually’ (p. 168). Some women also found that exercises could be difficult to engage in due to the religious dress code. One stated, ‘it was a little bit of a problem for the full cover, hijab wearing women’ and another that ‘it stressed me out a little because I had to cover my face the whole time which caused difficulties breathing and seeing’. (p. 168). However, designing programmes according to gender preferences might not always be appropriate. Greene et al. (2022) ‘described the importance of including and involving men’ as their programmed specifically focussed on ‘on efforts to reduce gender-based violence’ (p. 168).

#### Theme 3: Accessibility

The accessibility of MHPSS programmes to local recipients was a key theme in nine studies (Tol et al., 2018; Yassin et al., 2018; Doğan et al., 2019; Hamid et al., 2020; Kerbage et al., 2020; Mitchell-Gillespie et al., 2020; Akhtar et al., 2021; El-Khani et al., 2021; Fine et al., 2021). Sub-themes included i) language in which the programme was delivered compared to that of recipients, ii) the lack of literacy of programme recipients and iii) the location of services.

### Language

Language can be a significant barrier when delivering MHPSS programmes to displaced populations in LMICs, limiting accessibility. Four studies addressed the important role of language in the delivery of MHPSS interventions, with two considering the perspectives of intervention recipients (Tol et al., 2018; Doğan et al., 2019) and two considering the perspectives of mental health professionals delivering the intervention (Hamid et al., 2020; Mitchell-Gillespie et al., 2020). From these studies, both parties concurred that effective communication in languages understood by MHPSS recipients is essential for the accessibility of programmes. In Doğan et al. (2019), Syrian refugees revealed that their access to mental health services was limited as there was ‘only one Arabic-speaking doctor’, and when the doctor was unavailable, they ‘could not get prescriptions’ (p. 678). The lack of Arabic-speaking staff also meant that they ‘could not make appointments’ and had hindered access to ‘tests, medical imaging and medical reports’ (p. 676). In a similar vein, Tol et al. (2018) revealed a demand for MHPSS materials, including ‘audio-recording(s), illustrated manual(s) and worksheet(s)’, to be available in ‘different languages’ so as to cater to recipients ‘from different ethnic tribes’ (p. 9). The inability to do so would likely lead to the recipient’s discomfort and incompletion of MHPSS programmes, impeding accessibility. Recipients’ perceptions of language as a barrier to accessibility are also supported by CBR workers’ views in Mitchell-Gillespie et al. (2020). They determined the ‘lack of Arabic interface’ in CBR telehealth services as a major barrier to access, recommending a ‘built-in translation service’ for the accessibility of ‘participants not speaking the same language’ (p. 9). Correspondingly, Hamid et al. (2020) support the notion of language as a facilitator for accessibility through Syrian mental health professionals’ perception that their proficiency in ‘Syrian dialects’ enabled them to understand their clients’ ‘cultural, religious, political and social contexts’ and provide ‘words of comfort appropriately’ (p. 4).

### Literacy

Like language, illiteracy was also portrayed as a barrier to accessing MHPSS programmes in three studies (Tol et al., 2018; Kerbage et al., 2020; Fine et al., 2021). This was especially noticeable for programmes involving the use of materials. Fine et al. (2021) reported that MHPSS materials for the EASE intervention, characterised by ‘graphics and stories’, were generally regarded as ‘easy to understand, and accessible to non-literate participants’ (p. 7). However, there was still feedback about challenges for those with literacy issues, putting forth the need for additional efforts to cater to the lack of literacy among Burundian refugees. Tol et al. (2018) presented a similar issue with the illustrated manual and worksheets used in SH+, whereby intervention facilitators mentioned that participants who ‘were unable to read and write’ had to ‘take their books to their children or neighbours to read it for them’ (p. 9), establishing a connection between illiteracy and reduced accessibility. This connection is elucidated in Kerbage et al. (2020) in a different manner. Mental health professionals, including psychiatrists, perceived low levels of literacy as an indication that intervention recipients were ‘ignorant’ and ‘not educated’ (p. 5), and were convinced that illiteracy accounted for the latter’s ‘resistance to mental health treatment’ (p. 6). These negative attitudes towards illiteracy insinuate that such services were not friendly or accessible to non-literate individuals.

### Location/reach

The theme of location or site of MHPSS delivery was explored in six studies (Akhtar et al. (2021), Doğan et al., 2019, El-Khani et al., 2021, Fine et al., 2021, Mitchell-Gillespie et al., 2020, Yassin et al., 2018). All studies concurred that location plays a significant role in determining participants’ accessibility to the interventions. Fine et al. (2021) found that the appropriate ‘distance and location of sessions’ was one of the factors contributing to the ‘feasibility’ of the EASE (p. 6), while in El-Khani et al. (2021), location was recognised by a facilitator as having an influence on the reach of the Strong Families intervention. The facilitator thus recommended also implementing the intervention in other reception centres to allow ‘everyone’ to benefit (pp. 13–14). Yassin et al. (2018) affirm a similar notion through an MHPSS provider attributing regular attendance at ‘follow-up appointments’ to the centre’s accessibility and ‘close proximity to the camp’, which meant that ‘travel time and cost were not a challenge’ for recipients (p. 390). Likewise, Mitchell-Gillespie et al. (2020), CBR workers and managers, recognised that CBR delivered through telehealth would enable ‘more people to use such services’, suggesting that access and reach were confined by physical geographical location (p. 10–11).

In the same way, delivery sites that were situated far away from recipients’ residence impeded access due to their inability to afford transportation costs. EASE participants in Akhtar et al. (2021) cited ‘financial concerns related to initial cost of transportation to EASE locations’ as one of the ‘greatest barriers to attendance’ (p. 8). Recipients in Doğan et al. (2019) echo similar views, finding taxi rides to the hospital ‘economically challenging’, but were also faced with limited options as ‘public transport is difficult’ (p. 674).

## Discussion

Addressing issues related to the acceptability and accessibility of MHPSS programmes is key to their successful delivery and uptake (Dickson and Bangpan, 2018). Evidence on acceptability has provided important insights about how displaced populations are currently engaging with MHPSS programmes in LMICs. Similarly, issues related to accessibility have highlighted important themes about the practical barriers and facilitators to utilising programmes.

Stigma as a result of cultural and gender norms were key factors influencing the acceptability of MHPSS programmes. The stigma associated with needing and receiving help for mental health issues is evidenced across cultures and intersects with gender (Elshamy et al., 2023; Khatib et al., 2023). This review concurs with existing evidence to suggest that both ‘self-stigma’ (e.g., internalised sense of shame or feeling devalued due to mental health issues) and ‘societal stigma’ (e.g., whereby communities may stigmatise individuals with mental health issues) can impede the uptake of services (Bawadi et al., 2022; Abo-Rass et al., 2023). While mental health issues affect both men and women, traditional gender roles may prevent men from seeking help or discussing their emotional struggles as openly as women due to the stigma attached with doing so (Khatib et al., 2023). Cultural and gendered beliefs among displaced communities may stigmatise mental health issues, viewing them as personal failures or signs of weakness (Elshamy et al., 2023). Gender-specific barriers were observed in responses to certain programme components. With men showing reluctance towards activities perceived as ‘less masculine’, while women expressed enthusiasm for activities emphasising quality time and gender-separated groups. Limited understanding and awareness about how displacement can impact mental health can also contribute to further stigma (Abo‐Rass et al., 2023).

Given the negative perceptions that can be associated with mental health, interventions that provide psychoeducation about the importance of and necessity for good mental health and psychosocial well-being could also be beneficial prior to and during implementation. This could also be informed by an understanding of how psychological distress is perceived and articulated across different population groups to support greater cultural and gender sensitivity of programming (Hamid et al., 2020). Promoting MHPSS in a positive light may assure local communities that addressing their mental health is an indication of strength rather than a source of shame, thereby reducing the levels of stigma associated with MHPSS services. Greater consideration should also be given to the aims and objectives of MHPSS to decide whether programme components need to be tailored along gendered lines or whether single or mixed sex programming is most appropriate. Targeting the inclusion of men in MHPSS programmes, could also support efforts to reduce any gender disparities in mental health outcomes and enhance family and community dynamics. Our synthesis has also shown that religion can play a fundamental role in how local communities engage with MHPSS programmes. Individuals were much more open to programmes that reflected their religious beliefs. Thus, it would be helpful to assess the extent to which MHPSS programmes reflect the values of the communities they are serving and whether they would benefit from embedding religious beliefs into core programme components to further enhance their receptivity.

The second theme, accessibility, contained three sub-themes: language, literacy and location/reach. For the purposes of this review, literacy referred to the capacity to read and write, whereas language proficiency denoted the ability to communicate effectively in a second language. While there can be overlap, it is important to note that when accessing programmes, individuals with limited literacy could also face challenges in understanding the written materials, including when in their own language. Thus, it is important for programme implementers to bear in mind that displaced populations may be limited by educational levels as well as linguistic, financial and familial constraints. As such, ensuring that the materials and services are made available in the appropriate language and medium (e.g., pictures and diagrams instead of words, audio methods of delivery etc.) for the target population is indispensable for the successful delivery of interventions. Engagement with programme materials at this level could also be coupled with training on sensitive care models to help avoid stereotypes of illiterate participants as “ignorant” and ‘resistant’ to treatment (Kerbage et al., 2020).

MHPSS programmes were often delivered in person and in close proximity to participants’ residence to increase ease of access and time constraints. However, this also raised issues of privacy and safety due to the visibility of programme participation (Akhtar et al., 2021). Further flexible programming (e.g., offering multiple timeslots or multiple locations) could help in accommodating these concerns, including pressing familial commitments the displaced populations may have. Finally, enabling displaced populations to access the delivery site with ease, through providing transportation services to and from the delivery sites or a transportation stipend, might be another aspect to consider in enhancing the accessibility of the interventions. Delivering services through information and communication technology might also be an option to save travel time and costs. However, this may largely be dependent on the availability of electronic devices and a stable internet connection. Our findings echo ongoing calls for pragmatic programming that considers the wider socioeconomic context of delivery of displaced population (Jannesari et al., 2021).

### Strengths and limitations

This review builds on previous qualitative and mixed method approaches to produce a robust and transparent approach to evidence synthesis. By synthesising evidence on similar populations and interventions across a diverse set of research objectives, we were able to identify common themes and patterns within a complimentary but varied data set. However, in doing so, we also found that studies using different methods, measures and frameworks to evaluate processes can make it difficult to draw meaningful comparisons between them. Similarly, including data drawn from outcome evaluations ensured that we captured important insights about the safety and feasibility of programmes. However, as noted by Nemiro et al. (2022), it is important to acknowledge that in less controlled settings, the implementation of MHPSS interventions may differ considerably, potentially resulting in divergent participant experiences compared to those observed within a more tightly controlled environment required for evaluations, such as RCTS.

Although this review has synthesised the perspectives and experiences of displaced populations receiving MHPSS interventions and providers of those programmes, due to lack of resources, we were unable to consult with key stakeholders to explore if this research resonates with their concerns and experiences or consider implications of the findings; future research will benefit from such engagement. Studies conducted in languages other than English and published prior to 2018 could also provide insights not included in this review. Nevertheless, in light of these limitations, we were able to produce a comprehensive synthesis that explores key delivery mechanisms potentially contributing to the success or failure of MHPSS programmes targeting displaced populations.

## Conclusion

This review synthesised evidence on the process to gain insight into factors influencing the delivery and receipt of MHPSS programmes for displaced populations in LMICs. Based on our findings, it is recommended that future MHPSS programmes should address issues of accessibility and acceptability that are specific to local contexts to ensure successful uptake and retention of MHPSS programmes. Attention should be paid to designing programmes that account for existing gender and cultural norms to limit stigmatisation associated with mental health and increase the sensitivity and relevance of programme content to target populations. Consideration should also be given to whether programmes can be flexible in timing, location and language, in order to maximise their reach. Future programme design and evaluation would also benefit from stakeholder engagement prior to commencement to ensure that efforts to achieve cultural adaptivity remain a priority.

## Supporting information

Dickson et al. supplementary material Dickson et al. supplementary material

## Data Availability

The authors confirm that the data supporting the findings of this study are available within the article, references and/or its Supplementary Materials.

## Supplementary material

The supplementary material for this article can be found at http://doi.org/10.1017/gmh.2024.56.
