# Supplementary material for: Mental health and psychosocial support programmes for displaced populations in low- and middle-income countries (LMICs): A systematic review of process, perspectives and experiences
Source: Glob Ment Health (Camb). 2024 May 6;11:e62. doi: 10.1017/gmh.2024.56 (PMC11106547; doi:10.1017/gmh.2024.56)
Supplement: Dickson et al. supplementary material [file S2054425124000566sup001.docx]

**Supplementary material**

**PRISMA checklist**

| Section/ topic |  | Checklist item | Reported |
| --- | --- | --- | --- |
| Title | 1 | Identify the report as a systematic review, meta-analysis, or both. | P |
| Absact | | | |
| Structured summary | 2 | Provide a structured summary including, as applicable: background; objectives; data sources; study eligibility criteria, participants, and interventions; study appraisal and synthesis methods; results; limitations; conclusions and implications of key findings; systematic review registration number. | P |
| Introduction | | | |
| Rationale | 3 | Describe the rationale for the review in the context of what is already known. | P |
| Objectives | 4 | Provide an explicit statement of questions being addressed with reference to participants, interventions, comparisons, outcomes and study design (PICOS). | P |
| Methods | | | |
| Protocol and registration | 5 | Indicate if a review protocol exists, if and where it can be accessed (e.g. web address), and, if available, provide registration information including registration number. | P |
| Eligibility criteria | 6 | Specify study characteristics (e.g. PICOS, length of follow-up) and report characteristics (e.g. years considered, language, publication status) used as criteria for eligibility, giving rationale. | P |
| Information sources | 7 | Describe all information sources (e.g. databases with dates of coverage, contact with study authors to identify additional studies) in the search and date last searched. | P |
| Search | 8 | Present full electronic search strategy for at least one database, including any limits used, such that it could be repeated. | P |
| Study selection | 9 | State the process for selecting studies (i.e. screening, eligibility, included in systematic review, and, if applicable, included in the meta-analysis). | P |
| Data collection process | 10 | Describe method of data extraction from reports (e.g. piloted forms, independently, in duplicate) and any processes for obtaining and confirming data from investigators. | P |
| Data items | 11 | List and define all variables for which data were sought (e.g. PICOS, funding sources) and any assumptions and simplifications made. | P |
| Risk of bias in individual studies | 12 | Describe methods used for assessing risk of bias of individual studies (including specification of whether this was done at the study or outcome level), and how this information is to be used in any data synthesis. | P |
| Summary measures | 13 | State the principal summary measures (e.g. risk ratio, difference in means). | n/a |
| Synthesis of results | 14 | Describe the methods of handling data and combining results of studies, if done, including measures of consistency (e.g. I^2^) for each meta-analysis. | P |
| Risk of bias across studies | 15 | Specify any assessment of risk of bias that may affect the cumulative evidence (e.g. publication bias, selective reporting within studies). | n/a |
| Additional analyses | 16 | Describe methods of additional analyses (e.g. sensitivity or sub-group analyses, meta-regression), if done, indicating which were pre-specified. | n/a |
| Results | | | |
| Study selection | 17 | Give numbers of studies screened, assessed for eligibility and included in the review, with reasons for exclusions at each stage, ideally with a flow diagram. | P |
| Study characteristics | 18 | For each study, present characteristics for which data were extracted (e.g. study size, PICOS, follow-up period) and provide the citations. | P |
| Risk of bias within studies | 19 | Present data on risk of bias of each study and, if available, any outcome level assessment (see Item 12). | Supplement file 5 |
| Results of individual studies | 20 | For all outcomes considered (benefits or harms), present, for each study: (a) simple summary data for each intervention group (b) effect estimates and confidence intervals, ideally with a forest plot. | n/a |
| Synthesis of results | 21 | Present results of each analysis and meta-analysis done, including confidence intervals and measures of consistency. | P |
| Risk of bias across studies | 22 | Present results of any assessment of risk of bias across studies (see Item 15). | N/A |
| Additional analysis | 23 | Give results of additional analyses, if done (e.g. sensitivity or sub-group analyses, meta-regression [see Item 16]). | P |
| Discussion | | | |
| Summary of evidence | 24 | Summarize the main findings including the strength of evidence for each main outcome; consider their relevance to key groups (e.g. healthcare providers, users and policy makers). | P |
| Limitations | 25 | Discuss limitations at study and outcome level (e.g. risk of bias), and at review-level (e.g. incomplete retrieval of identified research, reporting bias). | P |
| Conclusions | 26 | Provide a general interpretation of the results in the context of other evidence, and implications for future research. | P |
|  | | | |
| Funding | 27 | Describe sources of funding for the systematic review and other support (e.g. supply of data); role of funders for the systematic review. | Acknowledgement |

**METHODS:**

**Example of search strategy**

**Medline search**

| 1. exp Clinical Trials as Topic/ or controlled trial*.mp. or exp Randomized Controlled Trials as Topic/ |  |
| --- | --- |
| 2. Interrupted time series analysis.mp. or exp Interrupted Time Series Analysis/ |  |
| 3. (Controlled before and after stud*).mp. [mp=title, abstract, original title, name of substance word, subject heading word, keyword heading word, protocol supplementary concept word, rare disease supplementary concept word, unique identifier] |  |
| 4. Pragmatic clinical trial*.mp. |  |
| 5. program evaluation.mp. or exp Program Evaluation/ |  |
| 6. exp Pilot Projects/ or pilot scheme*.mp. |  |
| 7. Outcome evaluation.mp. or exp Evaluation Studies as Topic/ |  |
| 8. Pilot stud*.mp. |  |
| 9. exp Feasibility Studies/ or Feasibility stud*.mp. |  |
| 10. Effectiveness intervention*.mp. |  |
| 11. exp "Outcome and Process Assessment (Health Care)"/ or exp "Outcome Assessment (Health Care)"/ or Outcome assessment.mp. |  |
| 12. Process assessment.mp. |  |
| 13. Control group*.mp. |  |
| 14. comparison group*.mp. |  |
| 15. Comparison stud*.mp. |  |
| 16. Repeated measure*.mp. |  |
| 17. Performance assessment.mp. |  |
| 18. Cross over trial*.mp. |  |
| 19. exp Double-Blind Method/ |  |
| 20. Quasi experiment*.mp. |  |
| 21. policy experiment*.mp. |  |
| 22. Natural experiment*.mp. |  |
| 23. Social experiment*.mp. |  |
| 24. 1 or 2 or 3 or 4 or 5 or 6 or 7 or 8 or 9 or 10 or 11 or 12 or 13 or 14 or 15 or 16 or 17 or 18 or 19 or 20 or 21 or 22 or 23 |  |
| 25. armed conflict*.mp. |  |
| 26. post conflict*.mp. |  |
| 27. conflict affected.mp. |  |
| 28. mass conflict*.mp. |  |
| 29. War/ or exp War Crimes/ or war.mp. |  |
| 30. conflict-related.mp. |  |
| 31. civil war.mp. |  |
| 32. ('war-exposed' or 'war-affected').mp. [mp=title, abstract, original title, name of substance word, subject heading word, keyword heading word, protocol supplementary concept word, rare disease supplementary concept word, unique identifier] |  |
| 33. postwar.mp. |  |
| 34. postconflict*.mp. |  |
| 35. Displacement.mp. or exp "Displacement (Psychology)"/ |  |
| 36. exp Refugees/ or refugee*.mp. |  |
| 37. Mass killing.mp. |  |
| 38. Genocide.mp. or exp Genocide/ |  |
| 39. Disaster*.mp. or exp Disasters/ |  |
| 40. Natural disaster*.mp. |  |
| 41. Earthquakes/ or Earthquake*.mp. |  |
| 42. Typhoon*.mp. |  |
| 43. exp Droughts/ or Drought*.mp. |  |
| 44. exp Floods/ or Flood*.mp. |  |
| 45. Industrial disaster*.mp. |  |
| 46. Political violence.mp. |  |
| 47. exp Relief Work/ or Humanitarian.mp. |  |
| 48. Hurricane.mp. or exp Cyclonic Storms/ |  |
| 49. displaced population*.mp. |  |
| 50. displaced person.mp. |  |
| 51. mass adversity.mp. |  |
| 52. Industrial accident*.mp. |  |
| 53. exp Volcanic Eruptions/ or Volcano*.mp. |  |
| 54. Landslide*/ or landslide*.mp. |  |
| 55. Avalanche*.mp. or exp Avalanches/ |  |
| 56. exp Tsunamis/ or Tsunami*.mp. |  |
| 57. Storm surge*.mp. |  |
| 58. Tornado*.mp. |  |
| 59. Cyclone*.mp. |  |
| 60. Infestation*.mp. |  |
| 61. Wildfire.mp. |  |
| 62. extreme temperature.mp. |  |
| 63. exp Terrorism/ or Terrorist attack*.mp. or exp Bioterrorism/ |  |
| 64. 25 or 26 or 27 or 28 or 29 or 30 or 31 or 32 or 33 or 34 or 35 or 36 or 37 or 38 or 39 or 40 or 41 or 42 or 43 or 44 or 45 or 46 or 47 or 48 or 49 or 50 or 51 or 52 or 53 or 54 or 55 or 56 or 57 or 58 or 59 or 60 or 61 or 62 or 63 |  |
| 65. Mental health*.mp. or exp Mental Health/ |  |
| 66. exp Mental Disorders/ or psychosocial.mp. or exp Depressive Disorder/ or exp Psychosocial Deprivation/ or exp Adaptation, Psychological/ or exp Social Adjustment/ or exp Stress, Psychological/ |  |
| 67. exp Psychiatric Somatic Therapies/ or psychiatric.mp. or exp Social Work, Psychiatric/ |  |
| 68. exp Psychotherapy, Rational-Emotive/ or exp Psychotherapy/ or exp Psychotherapy, Multiple/ or exp Psychotherapy, Group/ or psychotherapy.mp. or exp Psychotherapy, Brief/ or exp Psychotherapy, Psychodynamic/ |  |
| 69. exp Depression/ or exp Cognitive Therapy/ or exp Stress Disorders, Post-Traumatic/ or Psychological treatment*.mp. or exp Behavior Therapy/ |  |
| 70. Mental health service*.mp. or exp Mental Health Services/ |  |
| 71. Social support.mp. or exp Social Support/ |  |
| 72. exp Anxiety Disorders/ or Cognitive Behavioural Therap*.mp. |  |
| 73. Community-based psychosocial support.mp. |  |
| 74. exp Counseling/ or Counselling.mp. |  |
| 75. counseling.mp. or Counseling/ |  |
| 76. Cognitive processing therap*.mp. |  |
| 77. exp Art Therapy/ or Creative arts.mp. |  |
| 78. Debriefing.mp. or exp Crisis Intervention/ |  |
| 79. Economic support.mp. |  |
| 80. Exposure therap*.mp. or exp Implosive Therapy/ |  |
| 81. (Eye movement Desensitization and Reprocessing).mp. [mp=title, abstract, original title, name of substance word, subject heading word, keyword heading word, protocol supplementary concept word, rare disease supplementary concept word, unique identifier] |  |
| 82. Eclectic.mp. |  |
| 83. Group therap*.mp. |  |
| 84. Interpersonal therap*.mp. |  |
| 85. Grief Intervention*.mp. |  |
| 86. Family therap*.mp. or Family Therapy/ |  |
| 87. family-based intervention*.mp. |  |
| 88. Narrative exposure therap*.mp. |  |
| 89. Music therap*.mp. or exp Music Therapy/ |  |
| 90. Psychological intervention*.mp. |  |
| 91. Psychosocial care intervention*.mp. |  |
| 92. exp Relaxation/ or Relaxation.mp. or exp Relaxation Therapy/ |  |
| 93. Preventive psychosocial intervention*.mp. |  |
| 94. Psychodynamic therap*.mp. |  |
| 95. Skill based group*.mp. or exp Health Education/ |  |
| 96. Safe space.mp. |  |
| 97. psychoeducation.mp. |  |
| 98. Trauma focused intervention*.mp. |  |
| 99. Thought field therap*.mp. |  |
| 100. (Dance and movement therap*).mp. [mp=title, abstract, original title, name of substance word, subject heading word, keyword heading word, protocol supplementary concept word, rare disease supplementary concept word, unique identifier] |  |
| 101. Prolonged exposure therap*.mp. |  |
| 102. School-based.mp. |  |
| 103. Stress Inoculation Therap*.mp. |  |
| 104. KIDNET.mp. |  |
| 105. exp Psychophysiologic Disorders/ or Specialised psychotherapeutic intervention.mp. |  |
| 106. Interpersonal psychotherapy.mp. |  |
| 107. Testimony Therap*.mp. |  |
| 108. Trauma healing.mp. |  |
| 109. Reconciliation.mp. |  |
| 110. Psychopharmacological treatment*.mp. |  |
| 111. Physiotherapy.mp. |  |
| 112. Psychological care.mp. |  |
| 113. exp Home Care Services/ or exp Self Care/ |  |
| 114. (Sport and recreation).mp. [mp=title, abstract, original title, name of substance word, subject heading word, keyword heading word, protocol supplementary concept word, rare disease supplementary concept word, unique identifier] |  |
| 115. Case management.mp. or exp Case Management/ or exp "Referral and Consultation"/ |  |
| 116. exp Human Rights/ |  |
| 117. Legal services.mp. |  |
| 118. exp Vocational Education/ or exp Rehabilitation, Vocational/ or Vocational training.mp. |  |
| 119. Mentoring.mp. |  |
| 120. exp Community Mental Health Services/ or community oriented public mental health service*.mp. |  |
| 121. Resettlement assessment.mp. |  |
| 122. Outreach.mp. |  |
| 123. exp Self-Help Groups/ |  |
| 124. Psychotherapeutic intervention*.mp. |  |
| 125. Psychological first Aid.mp. |  |
| 126. ('implosive therap*' or flooding therap*' or 'imaginal floodings').mp. [mp=title, abstract, original title, name of substance word, subject heading word, keyword heading word, protocol supplementary concept word, rare disease supplementary concept word, unique identifier] |  |
| 127. 65 or 66 or 67 or 68 or 69 or 70 or 71 or 72 or 73 or 74 or 75 or 76 or 77 or 78 or 79 or 80 or 81 or 82 or 83 or 84 or 85 or 86 or 87 or 88 or 89 or 90 or 91 or 92 or 93 or 94 or 95 or 96 or 97 or 98 or 99 or 100 or 101 or 102 or 103 or 104 or 105 or 106 or 107 or 108 or 109 or 110 or 111 or 112 or 113 or 114 or 115 or 116 or 117 or 118 or 119 or 120 or 121 or 122 or 123 or 124 or 125 or 126 |  |
| 128. 24 and 64 and 127 |  |
| 129. limit 128 to (english language and humans and yr="1980 -Current") |  |
| 130. Ethnography.mp. |  |
| 131. Content analysis.mp. |  |
| 132. Participant observation.mp. |  |
| 133. Field note*.mp. |  |
| 134. exp "Process Assessment (Health Care)"/ or Process evaluation.mp. or exp "Outcome and Process Assessment (Health Care)"/ |  |
| 135. (Process measure* or process assessment*).mp. [mp=title, abstract, original title, name of substance word, subject heading word, keyword heading word, protocol supplementary concept word, rare disease supplementary concept word, unique identifier] |  |
| 136. Ethnopsychology.mp. or exp Ethnopsychology/ |  |
| 137. exp Qualitative Research/ or exp Focus Groups/ or Focus group*.mp. |  |
| 138. (Qualitative method* or Qualitative stud*).mp. [mp=title, abstract, original title, name of substance word, subject heading word, keyword heading word, protocol supplementary concept word, rare disease supplementary concept word, unique identifier] |  |
| 139. (group interview* or in-depth interview* or one-to-one interview*).mp. [mp=title, abstract, original title, name of substance word, subject heading word, keyword heading word, protocol supplementary concept word, rare disease supplementary concept word, unique identifier] |  |
| 140. Mixed-methods.mp. |  |
| 141. Thematic synthesis.mp. |  |
| 142. thematic analysis.mp. |  |
| 143. qualitative analysis.mp. |  |
| 144. framework synthesis.mp. |  |
| 145. framework analysis.mp. |  |
| 146. Grounded theory.mp. or exp Grounded Theory/ |  |
| 147. (Grounded research or grounded stud*).mp. [mp=title, abstract, original title, name of substance word, subject heading word, keyword heading word, protocol supplementary concept word, rare disease supplementary concept word, unique identifier] |  |
| 148. Constant comparative.mp. |  |
| 149. Theoretical saturation.mp. |  |
| 150. realist.mp. |  |
| 151. Constructionist.mp. |  |
| 152. (Pragmatism or realism).mp. [mp=title, abstract, original title, name of substance word, subject heading word, keyword heading word, protocol supplementary concept word, rare disease supplementary concept word, unique identifier] |  |
| 153. exp Feminism/ or Feminis*.mp. |  |
| 154. Social construction.mp. |  |
| 155. stakeholder view*.mp. |  |
| 156. acceptability.mp. |  |
| 157. affordability.mp. |  |
| 158. accessibility.mp. |  |
| 159. Implementation science.mp. |  |
| 160. exp Community-Based Participatory Research/ or Participatory research.mp. |  |
| 161. Intervention delivery.mp. |  |
| 162. fidelity.mp. |  |
| 163. Needs assessment.mp. or exp Needs Assessment/ |  |
| 164. 130 or 131 or 132 or 133 or 134 or 135 or 136 or 137 or 138 or 139 or 140 or 141 or 142 or 143 or 144 or 145 or 146 or 147 or 148 or 149 or 150 or 151 or 152 or 153 or 154 or 155 or 156 or 157 or 158 or 159 or 160 or 161 or 162 or 163 |  |
| 165. 64 and 127 and 164 |  |
| 166. limit 165 to (english language and humans and yr="1980 -Current") |  |
| 167. 129 or 166  **Websites searched**   - The World Bank: <http://www.worldbank.org/> - The Overseas Development Institute (ODI), including the Humanitarian Policy Group: <http://www.odi.org/programmes/humanitarian-policy-group> (HPG) and Humanitarian Practice Network: <http://odihpn.org/> (HPN) - Institute of Development Studies: <http://www.ids.ac.uk/> - International Development Research Centre: <http://www.idrc.ca/EN/Pages/default.aspx> - Active Learning Network for Accountability and Performance in Humanitarian Action (ALNAP): <http://www.alnap.org/> - Emergency Nutrition Network (Field Exchange): <http://www.ennonline.net/> - Evidence Aid: <http://www.evidenceaid.org/> - Feinstein International Center, Tufts University: <http://fic.tufts.edu/> - Enhanced Learning and Research for Humanitarian Assistance: <http://www.elrha.org/> - International Association of Professionals in Humanitarian Assistance and Protection: <https://phap.org/> - Humanitarian Accountability Partnership: <http://www.hapinternational.org/> (now CHS Alliance) - Network on Humanitarian Action: <http://nohanet.org/> - Harvard Humanitarian Initiative: <http://hhi.harvard.edu/> - Refugee Studies Centre, University of Oxford: <http://www.rsc.ox.ac.uk/> - European Commission Humanitarian Aid and Civil Protection Department (ECHO): <http://ec.europa.eu/echo/> - USAID Development Experience Clearinghouse (and related USAID sub-websites): <https://dec.usaid.gov/dec/home/Default.aspx> - ReliefWeb: <http://reliefweb.int/> - Oxfam Policy and Practice: <http://policy-practice.oxfam.org.uk/> - Mental Health and Psychosocial Support Network: <http://mhpss.net/> - UNHCR: <http://www.unhcr.org/cgi-bin/texis/vtx/home> - UNICEF: <http://www.unicef.org.uk/> - Asian Development Bank: <http://www.adb.org/about/main> - African Development Bank: <http://www.afdb.org/en/> - Inter-American Development Bank: <http://www.iadb.org/en/inter-american-development-bank,2837.html> - United Nations Office for the Coordination of Humanitarian Affairs (OCHA): <http://www.unocha.org/hina> - International Committee of the Red Cross (ICRC): <https://www.icrc.org/en> - Office of U.S. Foreign Disaster Assistance (OFDA), USAID: <https://www.usaid.gov/who-we-are/organization/bureaus/bureau-democracy-conflict-and-humanitarian-assistance/office-us> |  |

**Exclusion Criteria**

| Systematic Review Exclusion criteria: 1-6* (Bangpan et al. 2016) | | |
| --- | --- | --- |
| Criteria | | Rationale |
| 1. Date | not published after 1980 | We initially included studies published in or after 1980, as this is when MHPSS services began to become more widely available in humanitarian contexts. |
| 1. Participants | investigating populations who are a) military personnel or b) those working in humanitarian emergency contexts | We were interested in populations affected by humanitarian emergencies, including both adults and young people in LMICs. However, we excluded effectiveness studies if the majority of participants were military personnel as they fell outside the remit of our review. |
| 1. Interventions | not delivering MHPSS interventions in the context of humanitarian emergencies or for populations affected by humanitarian emergencies in LMICs | We included programmes that sought to provide MHPSS programmes delivered in the context of humanitarian emergencies or for populations affected by humanitarian emergencies. We wanted to include a broad range of programmes and therefore based on the advice of our advisory group, we adopted the Inter-Agency Standing Committee's (IASC) definition of MHPSS as any programme which seeks to ‘protect or promote psychosocial well-being and/or prevent or treat mental disorders’20 (p. 11), but which fall outside the remit of treatment programmes that are solely based on medication and pharmacology. Humanitarian emergencies were also broadly defined as any natural or man-made emergencies, including both slow-onset and sudden crises. The policy customer of the review was DFID and the scope was set at LMIC, not high income countries. We decided to continue with this for the updated versions of the review. |
| 1. Study design | not conducting a process evaluation | To answer the review question on barriers to, and facilitators of, implementing and receiving MHPSS interventions, we included primary studies where the primary focus is on intervention implementation regardless of study design. E.g. we included studies that focused exclusively on the ‘process’ of interventions or reported process data alongside outcome evaluation data. Studies not collecting primary data (e.g. systematic reviews, protocols, ongoing studies, linked studies were excluded). |
| 1. Reporting data | not collecting and reporting process data on the implementation of an MHPSS intervention | Studies need to collect data on the process of delivering or receiving MHPSS programmes. Types of data included participants’ perspectives captured by open-ended questions (e.g. interviews) or closed questions (e.g. surveys). We aimed to capture people’s experiences by reporting direct quotes by participants; author descriptions, either in narrative or numerical form; or authors’ conclusions. We excluded studies where the only data reported is ‘perception of impact’ and did not report on, delivery, feasibility, or other implementation processes. |
| 1. Language | Not written in English | Resource implications meant that we could only search for and include studies in English. |
| Qualitative Evidence Synthesis Exclusion Criteria: 7-8 (Dickson et al. 2024) | | |
| 7. Population | Not displaced populations (see definition below) | For this update of the review question on process we decided to narrow the focus to displaced populations. See definition used below. |
| 8. Date limit | not published in the last ten years (e.g. prior to 2013) | To ensure we included data relevant to current contexts we limited the studies to the last ten years. |

**Exclude 10: Definition of Displaced Populations:**

We adopted the the UN Refugee Agency (UNHCR), which are derived from the 1951 Convention on the Status of Refugees (UNHCR 2019).

- Refugee: a person who, owing to a well-founded fear of being persecuted for reasons of race, religion, nationality, membership of a particular social group, or political opinion, is outside the country of his nationality, and is unable to or, owing to such fear, is unwilling to avail himself of the protection of that country.
- Asylum seeker: an individual who is seeking asylum, but whose claim has not yet been finally decided on.
- Internally displaced persons: persons or groups of persons who have been forced or obliged to flee or to leave their homes or places of habitual residence, in particular as a result of, or in order to avoid the effects of, armed conflict, situations of generalised violence, violations of human rights or natural or human-made disasters, and who have not crossed an internationally recognised border.

**Methods: Critical appraisal**

An overall judgement of study quality was made according to two key dimensions. First, a weight of high, medium or low was assigned according to the reliability of the study using criteria 1–4. Second, a weight of high, medium or low was assigned according to the usefulness of the findings in answering the review question using criteria 5–6. To be judged as ‘high’ quality on methodological reliability, studies needed to have taken steps to ensure rigour in at least three of the first four criteria. Studies were judged as ‘medium’ when scoring on only 2–3 criteria and ‘low’ when scoring on only one or none. To achieve a rating of high on usefulness in answering the review question, studies needed to achieve depth or breadth in their findings and use methods that enabled participants to express their views on implementing or engaging in programmes. Studies rated as a medium on usefulness only met either one of these criteria and studies rated low were judged to have met neither criterion.

**Findings**:

Table A: Quality of included studies

| **Short Title** | **Were steps taken to minimise bias and error/increase rigour in sampling?** | **Were steps taken to minimise bias and error/increase rigour in data collection?** | **Steps were taken to minimise bias and error/increase rigour in data analysis?** | **Findings were grounded in/supported by the data?** | **Reliability** | **There was good breadth and/or depth achieved in the findings?** | **The perspectives of people were privileged?** | **Usefulness** |
| --- | --- | --- | --- | --- | --- | --- | --- | --- |
| Akhtar (2021) | • Yes | • Yes | • Yes | • Yes | High | • Yes | • Yes | High |
| Bawadi et al. (2022) | • Yes | • Yes | • Yes | • Yes | High | • Yes | • Yes | High |
| Doğan (2019) | • Yes | • Yes | • Yes | • Yes | High | • Yes | • Yes | High |
| Doumit et al. 2020 | • Yes | • Yes | • Yes | • Yes | High | • Yes | • No | Medium |
| El-Khani et al. (2021) | • No | • Yes | • Yes | • Yes | Medium | • No | • Yes | Medium |
| Fine et al. (2021) | • Yes | • Yes | • Yes | • Yes | High | • Yes | • No | Medium |
| Greene et al. (2022) | • Yes | • Yes | • Yes | • Yes | High | • Yes | • No | Medium |
| Hamid et al. (2020) | • Yes | • Yes | • Yes | • Yes | High | • Yes | • Yes | High |
| Kerbage et al. (2020) | • Yes | • Yes | • Yes | • Yes | High | • Yes | • Yes | High |
| Miller et al. (2020) | • Yes | • Yes | • Yes | • Yes | High | • Yes | • Yes | High |
| Mitchell-Gillespie et al. (2020) | • No | • No | • Yes | • Yes | Medium | • Yes. | • Yes | High |
| Murray et al. (2018) | • Yes | • Yes | • Yes | • Yes | High | • Yes | • Yes | High |
| Powell et al. (2023) | • Yes | • Yes | • Yes | • Yes | High | • Yes | • Yes | High |
| Tol et al. (2018) | • Yes | • Yes | • No | • Yes | Medium | • Yes | • No | Medium |
| Yassin et al. | • Yes | • Yes | • Yes | • Yes | High | • Yes | • Yes | High |

Table B: Cultural Adaptation

| **Authors** | **Contextual adaptation of MHPSS programmes (direct quotes)** |
| --- | --- |
| Akhtar et al. 2021 | EASE was initially adapted for Syrians residing in Lebanon and further adapted for use in Jordan. Following adaptation, focus groups were conducted with stakeholders and EASE facilitators to ensure the manual was contextually appropriate for Syrian refugees p.5 |
| Bawardi et al. 2022 | Not applicable/reported – exploring existing mental health services in the region |
| Dogan et al. 2019 | Not applicable/reported - exploring existing mental health services in the region |
| Doumit et al. 2020 | For each session, the PI and therapist went over each session in Arabic (live translation) and translated the homework assignment from English to Arabic, live in class. |
| El-Khani et al. 2021 | The version of Strong Families used in this study was previously culturally adapted, translated and reviewed in Afghanistan and successfully piloted on Afghan families in three Afghan cities. The program was translated to Serbian for the purpose of training and implementation in Serbia. More information on the content of the sessions of the Strong Families program can be found on the UNODC website. P.6 |
| Fine et al. 2021 | Prior to implementation, EASE was adapted for use with this population, with changes made to ensure that the intervention would be culturally and contextually appropriate. The current formative study lays the groundwork for a subsequent definitive trial of EASE among this population.' P3: 'Instruments were selected based on relevance, appropriateness for adolescent populations, prior use in LMICs and conflict-affected settings, and psychometric properties. P.2 |
| Greene et al. 2022 | To improve feasibility for the refugee camp context, we built on a more recently developed session protocol of cognitive processing therapy for the Nguvu intervention p.2869-2870 |
| Hamid et al. 2020 | Not applicable/reported. Mental Health Services not specified. |
| Kerbage et al. 2020 | We adapted as needed for the participants’ comfort and let them establish the interview setting while trying to combine acting ethically with responding culturally to different conceptions of privacy. Previous research has shown in this regard that in some indigenous contexts, Euro-American ethical codes for informed consent and confidentiality may not always make sense for community-centered social groups p.4 |
| Miller et al. | In selecting content for the CSI, we adopted a culturally integrative approach, drawing on well-researched concepts and methods from diverse cultural contexts. For example, in the sessions on managing and reducing stress, we explore ways of stepping back from ‘thinking too much’ (Am bfakr kteer), which entails thinking constantly about one’s problems, and worrying persistently about the future. Am bfakr kteer is salient throughout the Middle East and Central Asia. It has strong parallels with the Buddhist emphasis on overthinking as a source of suffering and with the western concept of rumination, common to both depression and anxiety. We also address anger and frustration using the Arabic and Turkish concept of Asabi. To become Asabi literally means to ‘lose one’s nerve’, but the term refers to becoming aggressively irritable or angry in response to life stress. We selected these cultural idioms because they are salient in the communities where the CSI was being developed. Our identification of locally salient stress reactions and expressions of distress was informed by the extensive clinical and research experience of our team in the region, as well as a review of the relevant literature. At the same time, we sought to identify expressions of stress and distress that reflect psychological states and processes found transculturally (i.e. stress-related irritability, overthinking/ rumination), an important factor in making the intervention readily adaptable to other cultural contexts. The stress management techniques used in the CSI draw on mindfulness practices which originated in South and Southeast Asia, but which have been widely adapted for use in other cultural contexts. The various practices share a focus on helping participants recognize and disengage from stress-inducing thoughts, either by re-focusing their attention on their breath or on another activity such as walking, or by using calming visual imagery. We also incorporated simple relaxation exercises such as progressive muscle relaxation, as well as anger management techniques such as counting to 10 or taking a short walk, before reacting to an emotionally charged situation. To avoid religious connotations that might cause discomfort in the traditional Muslim communities where the CSI was developed, we avoided the term mindfulness, with its Buddhist roots, instead referring to all of the stress management activities simply as ‘relaxation exercises.’ Once a zero draft protocol was developed, it was circulated for review among colleagues in Gaza and Lebanon, where the intervention would subsequently be implemented, and among a group of experts in parenting interventions. Feedback was incorporated, and we then moved to the next phase, the use of an iterative process that was key to developing a culturally acceptable and feasible intervention. Phase 1: development of initial curriculum, implementation and practice run in Gaza P6: Phase 2: Revision, implementation, and practice run with Syrian refugees in Lebanon. P7: Phase 3: implementation and evaluation of the revised CSI in a pilot randomized controlled trial. P.3-4 |
| Mitchell-Gillespie et al. 2020 | The research team recorded the session type, topic, real-time adaptations, modifications and feedback from CBR workers after each session. Feedback that recommended changes in training or sessions was considered by the research team and either adapted for the next session or remained unchanged due to technological or procedural constraints. P.4 |
| Murray et al. (2018) | However, CETA-Youth was adapted for a child/adolescent focus and incorporated additional elements to address externalizing symptoms and to incorporate parental involvement in the child’s treatment. Changing the population focus to children, adding an element (i.e. parenting skills), and adding caregiver steps for each element (so caregivers could support their children) had potential to compromise lay counselors’ ability to learn the intervention and deliver it with fidelity. However, our results suggest that CETA-Youth can be delivered by lay providers when they receive active training, practice opportunities, and supervision (Murray et al., 2011). Counselors, with supervision from local supervisors, were able to choose elements, their order, and dosing to include caregivers and address a range of comorbid symptomatology. This is important because comorbidity is the norm in most populations (Weisz et al., 2015). P.9-10 |
| Powell et al. (2023) | The intervention was developed in English and reviewed by the Jordanian staff at RHAS who were all fluent in Arabic and English. RHAS staff identified any cultural mismatches between cognitive-informational and environmental dimensions of the intervention. Cognitive-informational dimensions include program modifications to ensure participants understand the content of program material, whereas environmental adaptations address the applicability of the structure and content of the intervention (Marsiglia & Booth, 2015). Environmental dimensions included a review of the intervention structure and composition to ensure alignment with cultural values and norms. For example, in the original design the team discussed separating males and females due to gender dynamics in the communities they worked. The Jordanian staff noted that while there may be gender differences, women were unable to walk to the clinic without a male escort, therefore, the team determined the pilot would be conducted with both males and females. Cognitive-informational adaptations included assessment of content for linguistic transferability and comprehension of material (Marsiglia & Booth, 2015). For example, the term ‘emotional wellness’, was not transferable into Arabic, therefore, it was translated into the Arabic term of ‘mental well-being’ (العاطفية السالمه(. P.162-163) |
| Tol (2018) | Initial development of the generic intervention took place in response to the crisis in Syria, with the generic version designed to be applicable, once adapted, to diverse socio-cultural settings. A need for further adaptations to SH+ was identiﬁed during the training of facilitators and by facilitators during the ﬁrst pilot groups. Training was provided to four female SH+ facilitators (selected to have completed secondary education; proﬁcient spoken Juba Arabic; and good English language skills) over 5 days by two authors (KC, FB) involved in the development of SH+. |
| Yassin et al. (2018) | Not applicable/not stated |
