# Supplementary material for: Mental health and psychosocial support programmes for displaced populations in low- and middle-income countries (LMICs): A systematic review of process, perspectives and experiences
Source: Glob Ment Health (Camb). 2024 May 6;11:e62. doi: 10.1017/gmh.2024.56 (PMC11106547; doi:10.1017/gmh.2024.56)
Supplement: Peer review history

# Editor report: Recommendation: Mental health and psychosocial support programmes for displaced populations in low- and middle-income countries (LMICs): A systematic review of process, perspectives and experiences — R0/PR2

**DOI:** 10.1017/gmh.2024.56.pr2

**Affiliations:** Program on Forced Migration and Health, Columbia University Mailman School of Public Health, United States

As noted by the reviewers, the manuscript makes an important contribution to the field. The reviewers have made some recommendations that I hope the authors will consider.

# Editor report: Decision: Mental health and psychosocial support programmes for displaced populations in low- and middle-income countries (LMICs): A systematic review of process, perspectives and experiences — R0/PR3

**DOI:** 10.1017/gmh.2024.56.pr3

**Affiliations:** London School of Hygiene & Tropical Medicine, United Kingdom of Great Britain and Northern Ireland

No accompanying comment.

# Editor report: Recommendation: Mental health and psychosocial support programmes for displaced populations in low- and middle-income countries (LMICs): A systematic review of process, perspectives and experiences — R1/PR5

**DOI:** 10.1017/gmh.2024.56.pr5

**Affiliations:** Program on Forced Migration and Health, Columbia University Mailman School of Public Health, United States

As noted by the reviewer, the revised version of the manuscript has addressed the previous concerns and incorporated many of the reviewer suggestions. Thank you to the authors for the careful consideration of these reviewer comments.

One reviewer has made a few additional suggestions, which are primarily editorial in nature. We hope the authors will consider revising their manuscript according to these suggestions.

# Editor report: Decision: Mental health and psychosocial support programmes for displaced populations in low- and middle-income countries (LMICs): A systematic review of process, perspectives and experiences — R1/PR6

**DOI:** 10.1017/gmh.2024.56.pr6

**Affiliations:** London School of Hygiene & Tropical Medicine, United Kingdom of Great Britain and Northern Ireland

No accompanying comment.

# Editor report: Recommendation: Mental health and psychosocial support programmes for displaced populations in low- and middle-income countries (LMICs): A systematic review of process, perspectives and experiences — R2/PR8

**DOI:** 10.1017/gmh.2024.56.pr8

**Affiliations:** Program on Forced Migration and Health, Columbia University Mailman School of Public Health, United States

Congratulations on this important manuscript!

# Editor report: Decision: Mental health and psychosocial support programmes for displaced populations in low- and middle-income countries (LMICs): A systematic review of process, perspectives and experiences — R2/PR9

**DOI:** 10.1017/gmh.2024.56.pr9

**Affiliations:** London School of Hygiene & Tropical Medicine, United Kingdom of Great Britain and Northern Ireland

No accompanying comment.
